# Preparation and Characterization of Electrostatically Crosslinked Polymer–Liposomes in Anticancer Therapy

**DOI:** 10.3390/ijms19061615

**Published:** 2018-05-30

**Authors:** Yi-Ting Chiang, Sih-Ying Lyu, Yu-Han Wen, Chun-Liang Lo

**Affiliations:** 1 School of Pharmacy, China Medical University, Taichung City 402, Taiwan; ytchiang@mail.cmu.edu.tw; 2 Department of Biomedical Engineering, National Yang Ming University, Taipei City 112, Taiwan; karenlu0228@gmail.com (S.-Y.L.); qooaz0815@gmail.com (Y.-H.W.)

**Keywords:** liposome, anticancer therapy, pH-sensitivity, polymer

## Abstract

pH-sensitive polymer–liposomes can rapidly release their payloads. However, it is difficult to simultaneously achieve stability and pH-responsiveness in the polymer–liposomes. In this study, stable and pH-sensitive crosslinked polymer–liposomes were fabricated through electrostatic interactions. The pH-sensitive copolymer methoxy poly(ethylene glycol)-*block*-poly(methacrylic acid)-cholesterol (mPEG-*b*-P(MAAc)-chol) and crosslinking reagent poly(ethylene glycol) with end-capped with lysine (PEG-Lys2) were synthesized and characterized. At physiological conditions, the pH-sensitive copolymers were anionic and interacted electrostatically with the cationic crosslinker PEG-Lys2, forming the electrostatically-crosslinked polymer–liposomes and stabilizing the liposomal structure. At pH 5.0, the carboxylic groups in mPEG-*b*-P(MAAc)-chol were neutralized, and the liposomal structure was destroyed. The particle size of the crosslinked polymer–liposomes was approximately 140 nm and the polymer–liposomes were loaded with the anticancer drug doxorubicin. At pH 7.4, the crosslinked polymer–liposomes exhibited good stability with steady particle size and low drug leakage, even in the presence of fetal bovine serum. At pH 5.0, the architecture of the crosslinked polymer–liposomes was damaged following rapid drug release, as observed by using transmission electron microscopy and their apparent size variation. The crosslinked polymer–liposomes were pH-sensitive within the endosome and in the human breast cancer cells MDA-MB-231, as determined by using confocal laser scanning microscopy. The intracellular drug release profiles indicated cytotoxicity in cancer cells. These results indicated that the highly-stable and pH-sensitive electrostatically-crosslinked polymer–liposomes offered a potent drug-delivery system for use in anticancer therapies.

## 1. Introduction

Liposomes have been employed in the pharmaceutical industries and approved for use as an anticancer therapy by the US Food and Drug Administration (FDA) for decades [1]. In the development of artificial liposomes, poly(ethylene glycol) (PEG) was successfully introduced into the surface of liposomes to form PEGylated liposomes or “stealth” liposomes [2]. PEGylated liposomes efficiently prolong the circulation time of liposomes, as PEG prevents the adhesion of serum proteins onto the liposomal surface [3,4]. The half-life of the conventional liposomes was reported to be 13.5 h, whereas the half-life of PEGylated liposomes was extended to 50 h in humans [5]. Although the long-circulating PEGylated liposomes have enhanced tumor accumulation and improved the anticancer efficiency of the delivered drugs [6,7], there is still one major drawback.

The most challenging limitation of the PEGylated liposomes is the slow drug release profiles [8]; in tumor cells or tissues, this may lead to a reduction in the anticancer efficiency of the PEGylated liposomes [9]. To facilitate anticancer drug release in cancer cells or lesions, polymers with the ability to specifically respond to the tumor environments; for example, pH-responsive or temperature-sensitive polymers, were designed and incorporated into the liposomal systems [10,11,12]. Among all tested systems, the pH-responsive polymer–liposomal system was considered to represent a promising approach because tumor tissues and endosomes or lysosomes during endocytosis were found to be slightly more acidic than the physiological conditions [13,14,15]. Several attempts have been reported [10,11,16,17,18,19,20,21,22,23,24,25]; notably, these mainly employed carboxylated polymers [11,13,18,19,20,24,26,27]. The carboxylated polymers were maintained in an anionic state at physiological conditions, but were protonated at lower pH values, leading to the hydrophobicity of the polymer–liposomes [11,19]. As an example of this, Yuba et al. integrated the carboxylated polymer poly(glycidol)s into the liposomal system. At lower pH values, the carboxylated poly(glycidol)s were protonated and transformed into hydrophobic polymers. The poly(glycidol)-liposomes were thereafter damaged below pH 7.0 [19]. T. Chen et al. have prepared carboxylated polymer poly(ethylacrylic acid)-liposomes. At pH below 6.0, the polymer–liposomes were destroyed, which released their payloads [28]. However, it is difficult to ensure both stability and pH-sensitivity of the carboxylated polymer–liposomes [29], probably owing to the detachment of the superficial polymers [30]. Lee et al. developed and fabricated a polymer-caged liposomal system to achieve both stability and pH-responsiveness for drug delivery. They prepared a pH-responsive poly(acrylic acid) liposomal system with crosslinkers inserted into the polymer–liposome complex, which formed covalent bonds to stabilize the liposomal structures [31]. Although the covalently crosslinked polymer–liposomes possessed high stability and pH-responsiveness, their elimination was difficult, owing to the higher molecular weight of the polymer molecular [32].

**Scheme 1:**
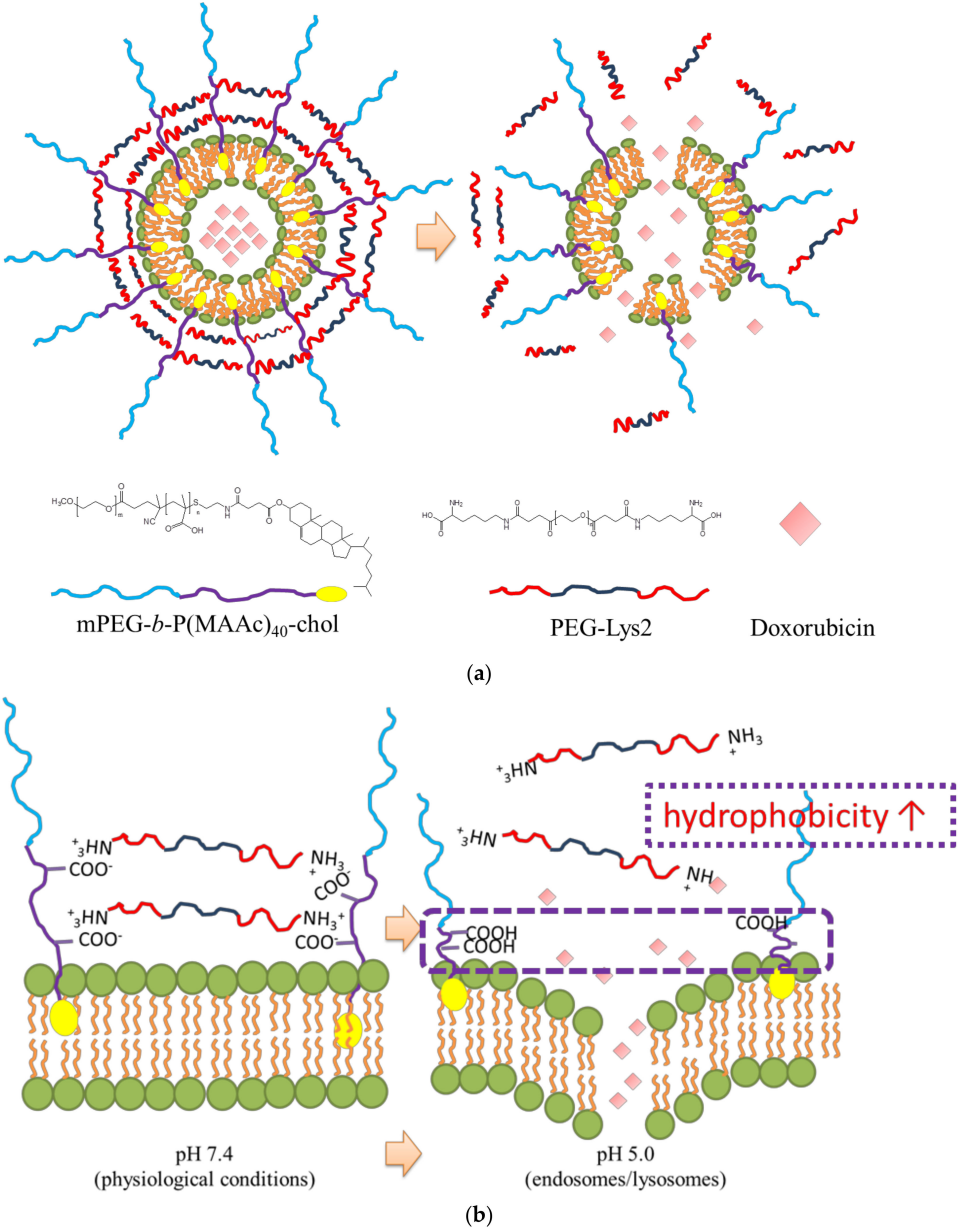
Concepts of the crosslinking polymer–liposomes (PC liposomes). (**a**) The crosslinking polymer–liposomes were composed of the pH-responsive copolymer mPEG-*b*-P(MAAc)_40_-chol, crosslinkers PEG-Lys2, and phospholipids; (**b**) At pH 7.4, the crosslinked polymer–liposomes were stabilized by the electrostatic forces between the anionic pH-responsive copolymers and the cationic crosslinkers. At pH 5.0, the protonation of the carboxylic groups occurred on the pH-responsive copolymers. The electrostatic forces were therefore destroyed, increasing the hydrophobicity of mPEG-*b*-P(MAAc)_40_-chol increased and releasing the anticancer drug doxorubicin.

In this study, we have designed and fabricated a novel, highly-stable, and pH-responsive polymer–liposome through electrostatic crosslinking. The polymer–liposomal systems were composed of a pH-responsive copolymer, methoxy poly(ethylene glycol)-*block*-poly(methacrylic acid)-cholesterol (mPEG-*b*-P(MAAc)-chol); a crosslinker, poly(ethylene glycol) end-capped with lysine (PEG-Lys2); and a phospholipid, dipalmitoylphosphatidylcholine (DPPC). At pH 7.4, the pH-responsive copolymers were inserted into the DPPC lipid bilayers, forming polymer-incorporated liposomes (PI liposomes). The PI liposomes were anionic because of the carboxylic acids in the mPEG-*b*-P(MAAc)-chol, whereas the crosslinkers were cationic owing to the amine groups of the end-capped lysine. The anionic pH-responsive copolymers mPEG-*b*-P(MAAc)-chol were crosslinked with the cationic crosslinker PEG-Lys2 via the electrostatic interaction at neutral pH, which strengthened the liposomal structures to produce crosslinked polymer–liposomes (PC liposomes). In acidic conditions, the protonation occurred on the carboxylic groups and led to a decrease in the anionic charges. The crosslinkers thereafter dissociated from the polymer–liposomal system. Simultaneously, the protonation also caused hydrophobicity, destroying the liposomal structures and releasing the payloads, as shown in Scheme 1. To the best of our knowledge, this is the first report of novel, electrostatically crosslinked liposomal structures. In our study, the pH-responsive copolymer mPEG-*b*-P(MAAc)-chol and the crosslinker PEG-Lys2 were synthesized and characterized. The physicochemical properties of the novel polymer–liposomes, such as particle size, zeta potential, and morphology, were investigated. Their stability and pH-responsiveness were also evaluated. In addition, the anticancer drug doxorubicin was encapsulated, and in vitro tests on internalization and cytotoxicity were also evaluated for future anticancer applications.

## 2. Results

### 2.1. Synthesis and Characterization of pH-Responsive Polymer and Crosslinkers

In this study, electrostatically crosslinked pH-responsive polymer–liposomes with were designed and fabricated. The liposomes were composed of phospholipids, pH-responsive copolymer (mPEG-*b*-P(MAAc)-chol), and crosslinkers (poly(ethylene glycol) end-capped with lysine (PEG-Lys2)). The pH-sensitive copolymers possessed carboxylic groups; at low pH values, these could be protonated to become hydrophobic [19].

The copolymer mPEG-*b*-P(MAAc)_40_-chol was synthesized by free radical polymerization using the macroinitiator methoxy poly(ethylene glycol) modified with 4,4′-azobis-(4-cyanopetanoic acid) (mPEG_2_-ABCPA), as previously reported [21]. The macroinitiator mPEG_2_-ABCPA, transfer reagent 2-amino-ethabethiol hydrochloride (AET-HCl), and monomer methacrylic acid (MAAc) were dissolved in ethanol, and polymerization was initiated at 70 °C. After 24 h, the copolymers of mPEG-*b*-P(MAAc)_40_-NH_2_ were obtained and purified by using ethyl ether. The chemical structure of mPEG-*b*-P(MAAc)_40_-NH_2_ was determined by using ^1^H NMR and FT-IR, as shown in Figure S1a,b. The ^1^H NMR spectrum of the mPEG-*b*-P(MAAc)_40_-NH_2_ in DMSO-*d*_6_ solvent is shown in Figure S1a; the hydrogen atoms in the methyl and methylene groups of the MAAc were at shifts of 0.8–1.0 and 1.6–2.0 ppm, respectively, and the hydrogen atoms on the ethylene groups in PEG were found at 3.5 ppm. These hydrogen atoms were used to calculate the repeating units, which were approximately 40 in number. In addition, the chemical shift at 7.85 ppm represented the hydrogen atoms in the amine group of the copolymer, which proved that the amine groups were successfully introduced into the copolymer via the chain transfer. The FT-IR spectrum supported the ^1^H NMR results. The C–O bonds in PEG were observed in the FT-IR spectrum at 1150 cm^−1^. The peak at 1500–1600 cm^−1^, which represented N–H bending, and the broad bands observed at 3500 cm^−1^, indicated that the terminal amine groups resulted from the chain transfer reagents. The strong vibrational peaks at 2800 cm^−1^ demonstrated the O–H bonds in the carboxylic acid groups of MAAc. Both spectra illustrated the successful fabrication of the intermediate copolymer mPEG-*b*-P(MAAc)_40_-NH_2_.

**Figure 1:**
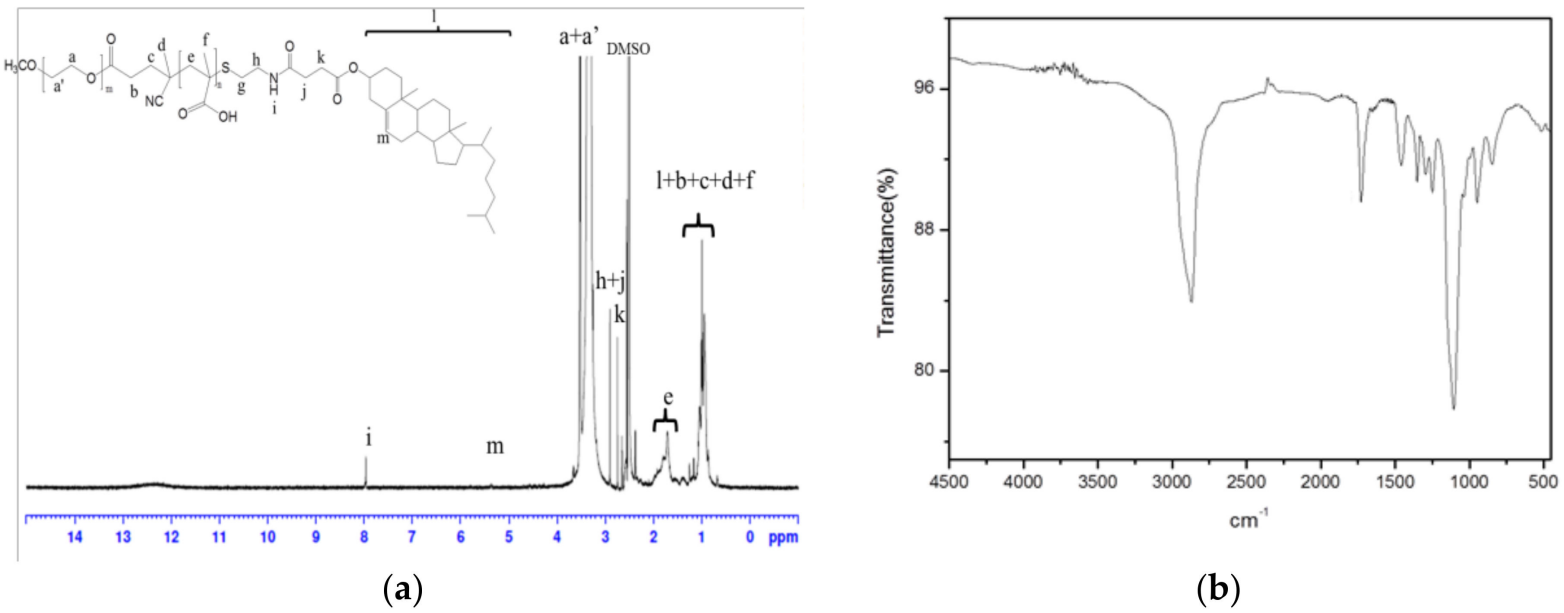
Characterization of the copolymers mPEG-*b*-P(MAAc)-chol. The chemical structures of the copolymers were characterized using the ^1^H NMR in DMSO-*d*_6_ (**a**) and FT-IR (**b**).

To provide the hydrophobic tails for insertion into the liposomal structure, cholesterol was introduced by using cholesterol-NHS ester (Chol-NHS ester) capping onto the amine (NH_2_) group of the copolymers, which completed the pH-responsive polymer mPEG-*b*-P(MAAc)_40_-chol. The mPEG-*b*-P(MAAc)_40_-NH_2_ was first dissolved with Chol-NHS ester in the acetone/methanol co-solvents. After 24 h at room temperature (25 °C), the copolymers were purified via ethyl ether precipitation to remove excess Chol-NHS ester. The obtained copolymers were characterized by using ^1^H NMR and FT-IR, shown respectively in Figure 1a,b. All experimental chemical shifts were assigned to corresponding hydrogen atoms of mPEG-*b*-P(MAAc)_40_-chol: 0.8–1 ppm for methyl groups (from MAAc); 1–2 ppm from cholesterol and –CH_2_– (from MAAc); 2.8–3.0 ppm for the succinic group (from Chol-NHS ester), and 3.5 ppm for ethylene groups of PEG (Figure 1a). The FT-IR spectra provided further evidence the obtained mPEG-*b*-P(MAAc)_40_-chol/one strong vibrational peak at 2750 cm^−1^ (OH carboxylic acid in MAAc); a peak at 1750 cm^−1^, which indicated a higher frequency of the ester bond owing to the ester groups from Chol-NHS ester; the peaks at 1600 and 3400 cm^−1^, assigned to the N–H bending and primary amine groups, respectively, disappeared, which indicated that the amine groups in mPEG-*b*fP(MAAc)_40_-NH_2_ were successfully conjugated with the Chol-NHS ester. Thus, the ^1^H NMR and FT-IR spectra demonstrated the successful preparation of mPEG-*b*-P(MAAc)_40_-chol.

**Figure 2:**
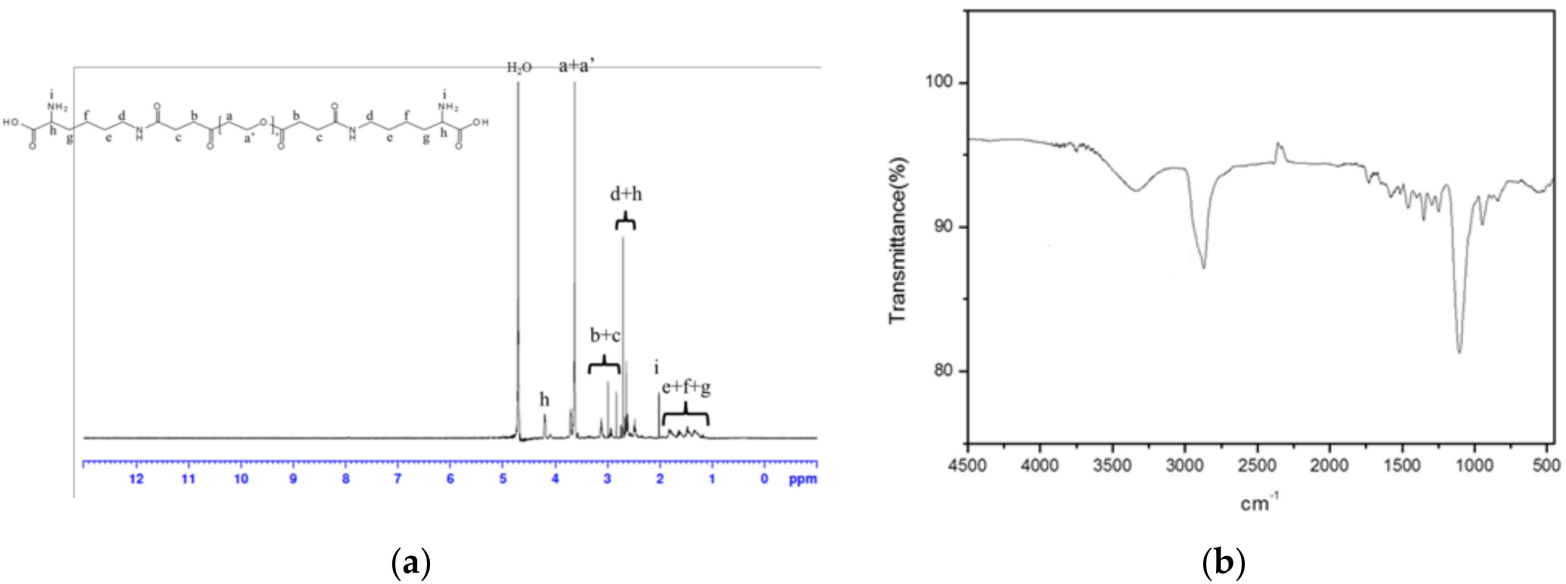
Characterization of the crosslinking reagents PEG-Lys2. The chemical structures of the copolymers were characterized by using ^1^H NMR in D_2_O (**a**) and FT-IR (**b**).

The crosslinker PEG-Lys2 was also prepared via an end-capping modification of PEG200. The hydroxyl group at the both ends of PEG200 was first modified into NHS ester, and the amine groups on the lysine were allowed to react further with the NHS ester groups to obtain PEG-Lys2. The chemical structure of PEG-Lys2 was also determined by ^1^H NMR and FT-IR, as shown in Figure 2a,b, respectively: the chemical shifts of the PEG-Lys2 toward the relevant hydrogen atoms, shown in the ^1^H NMR spectra in Figure 2a, can be described as follows: 1–2 ppm for the –CH2– from lysine, 2.6 and 2.9 ppm for the succinic groups, and 3.5–2.6 ppm for the ethylene groups from PEG; the FT-IR spectrum, presented in Figure 2b, revealed the functional groups in PEG-Lys2, with the C–O bonds mainly from PEG shown at 1150 cm^−1^, a strong vibrational peak at 2850 cm^−1^ was observed, which represented the OH from the carboxylic acid in the end-capped lysine, and the broad band at 3350 cm^−1^ apparently demonstrated the amine groups on the two end-capped lysines. Both ^1^H NMR and FT-IR results were indicative of the complete synthesis of the crosslinkers.

### 2.2. Preparation and Characterization of the Crosslinked Polymer–Liposomes

The synthesized pH-responsive copolymers mPEG-*b*-P(MAAc)_40_-chol and crosslinker PEG-Lys2 were further organized into pH-responsive crosslinked polymer–liposomes by using the phospholipid DPPC. The pH-responsive copolymers mPEG-*b*-P(MAAc)_40_-chol and phospholipid DPPC were first homogeneously prepared as thin films and subsequently hydrated. The hydrated thin films were forced into liposomal structure via probe sonication and extrusion. The cholesterol groups of the copolymers were inserted into the lipid bilayers through the hydrophobic forces during hydration and probe sonication, which formed the polymer-incorporated liposomes (PI liposomes). The crosslinker PEG-Lys2 was added into the PI liposomes. The cationic amine groups on PEG-Lys2 interacted electrostatically with the negatively-charged carboxylic groups to form the crosslinked polymer–liposomes (PC liposomes). In addition, DPPC liposomes, with and without the additional crosslinkers, were also prepared for further evaluation.

The physicochemical properties, including the hydrodynamic diameter and particle size distribution, were first evaluated by using dynamic laser scattering (DLS); the results are shown in Table 1. The particle sizes of the DPPC liposomes (DL) and the DPPC liposomes with additional crosslinkers (DCL) were 144.27 ± 7.49 and 151.10 ± 4.69 nm, respectively. The hydrodynamic diameters of polymer-incorporated liposomes (PI liposomes) were approximately 136 nm. Crosslinkers were added at an approximate molar ratio of 5–15% mole ratio, and electrostatically interacted with PI liposomes to form PC-A liposomes (5% molar ratio), PC-B liposomes (10% molar ratio), and PC-C liposomes (15% molar ratio). The particle sizes of all the PC liposomes were approximately 140 nm, which was a little larger than that of the PI liposomes. The particle dispersion index (PDI) for all liposomal samples was approximately 0.2. Among all samples, the DPPC liposomal samples, including DL and DCL, exhibited higher PDI values. The results indicated that the crosslinker did not have too much effect on the liposomal particle sizes and PDI.

### 2.3. Stability and pH-Responsiveness of the Crosslinked Polymer–Liposomes

**Figure 3:**
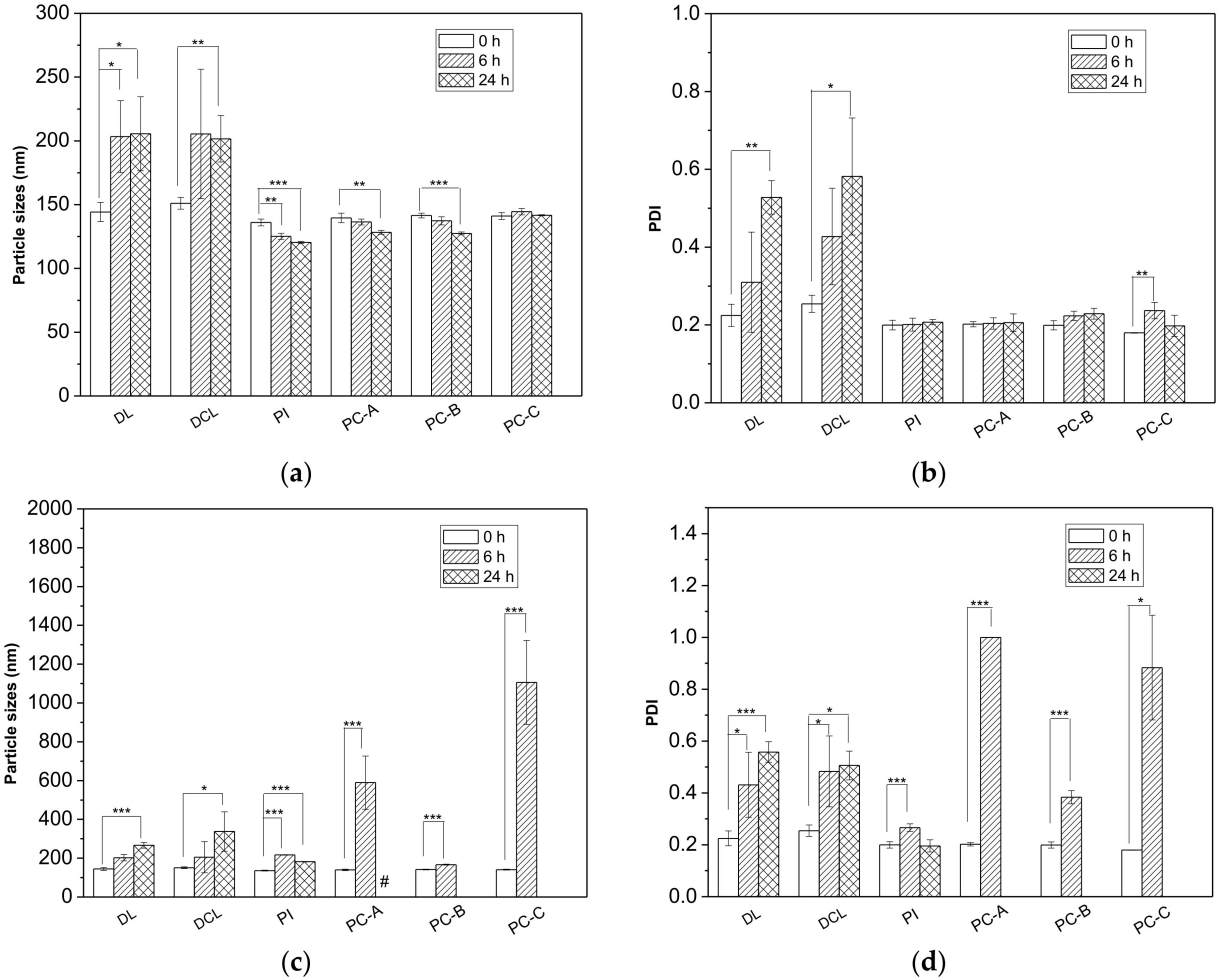
Stability and pH-responsive behavior of dipalmitoylphosphatidylcholine–liposomes (DPPC), polymer-incorporated liposomes (PI), and crosslinking polymer–liposomes (PC-A, PC-B, PC-C). Liposomal samples were incubated at 37 °C and pH 7.4, for stability assessment. After 6 and 24 h, the hydrodynamic diameters (**a**) and particle distributions (**b**) were determined by using dynamic laser scattering (DLS). The pH-responsive behavior was also studied through incubation of the liposomal samples at 37 °C and pH 5.0. After incubation for 6 and 24 h, the particle sizes (**c**) and distributions (**d**) were measured. The symbol “#” represents particle sizes undetectable by using DLS. Data were statistically analyzed using Student’s *t*-test. Significant differences have been indicated with asterisks (* *p* < 0.05; ** *p* < 0.001; *** *p* < 0.0005).

The stability of the DPPC, PI, and PC liposomes were evaluated and the additional concentration of the crosslinkers was optimized. Their stability was assessed using the particle size and distribution changes at predetermined times under simulated physiological conditions (37 °C, pH 7.4; the results are shown in Figure 3a,b.

The changes in particle size of the DPPC, PI, and PC liposomes (i.e., PC-A, PC-B, and PC-C liposomes) after 6 and 24 h incubation at 37 °C and pH 7.4 are shown in Figure 3a. The DL liposomes exhibited significant size increments within 6 h. After incubation for 24 h, DL and DCL increased in size by almost 1.5-fold. In addition, the PDI values increased from 0.2 to 0.5, as shown in Figure 3b, which indicated the heterogeneity of the DPPC liposomes. The incremental particle size and PDI values demonstrated the instability of the DL and DCL liposomes. The PI liposomes also exhibited particle size changes after 6 and 24 h of incubation. The particle sizes reduced over time. PI liposomes became 10% smaller owing to destabilization. No PC liposomes showed considerable alterations in particle size after 6 h of incubation, whereas after 24 h of incubation, the PC-A and PC-B liposomes showed huge particle size changes. A change of approximately 10% in particle size was seen, similar to that of the changes in the PI liposomes. For the PC-C liposomes, which contained the highest molar ratio of the crosslinkers, steady particle sizes were observed. Although the PDI of PC-C liposomes appeared to be altered after incubation for 6 h, the PDI values decreased after 24 h. From the results, it was concluded that PC-C liposomes possessed the greatest stability of the DPPC and polymer–liposomes, even though PC-C liposomes possessed slight dynamic non-homogeneity after incubation for 6 h.

The pH-responsiveness of the liposomes was also discussed in this study. The liposomal samples were placed in pH 5.0 at 37 °C. At a predetermined time, the particle size and distribution were measured to evaluate the pH-sensitivity; the lower pH environment was chosen to mimic the endocytosis process. The results are summarized in Figure 3c,d. The DL and DLC liposomes were approximately twice as large after incubation for 24 h, whereas the PDI values significantly increased from 6 h post-incubation. At pH 5.0, the DL liposomes were enlarged after incubation for 24 h. The PI liposomes and all PC liposomes were also incubated under the same conditions. After 6 and 24 h, PI liposomes and all PC liposomes exhibited major particle size alteration. After 24 h, the particle sizes of the PI liposomes were 33% greater, whereas all the particle sizes of the PC liposomes could not be precisely detected using DLS because of precipitation and aggregation. The significant particle size changes were attributable to the carboxylic groups on the copolymer mPEG-*b*-P(MAAc)_40_-chol. At pH 5.0, the carboxylic groups were protonated, which destroyed the liposomal structures.

**Figure 4:**
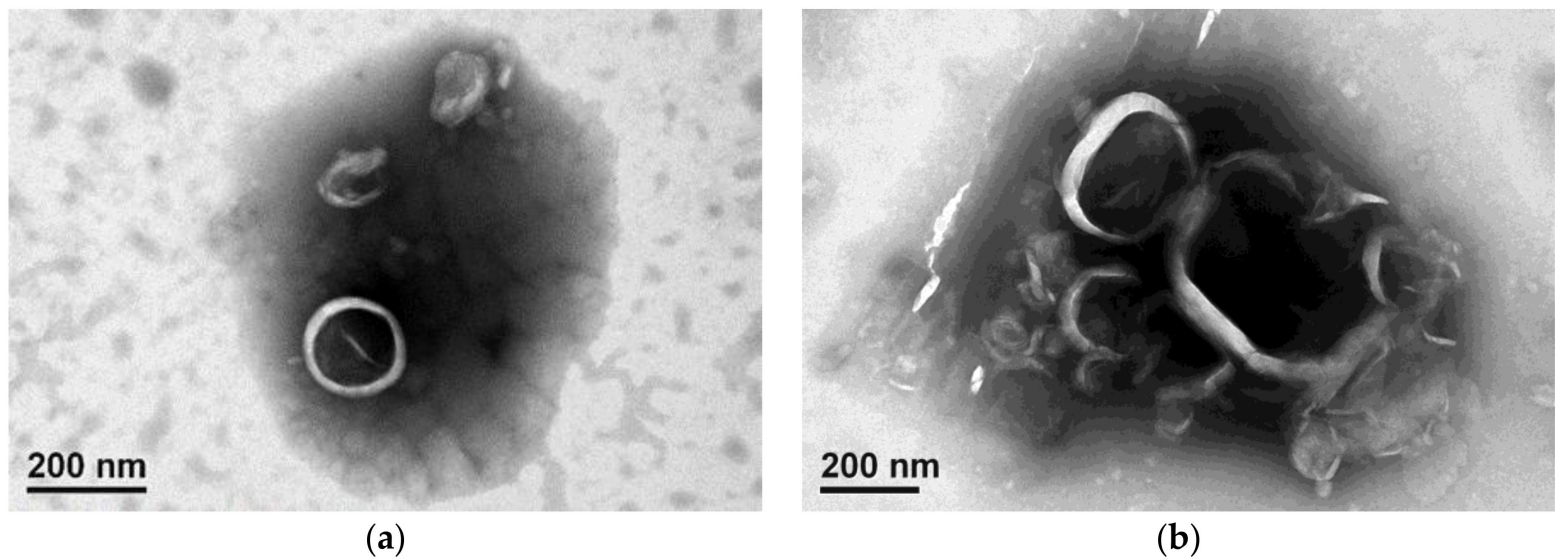
TEM images of the PC liposomes after incubation at pH 7.4 (**a**) and pH 5.0 (**b**) for 24 h. The polymer–liposome samples were dropped onto the copper grid and stained with 1 wt % phosphotungstic acid solution. The morphologies of the PC-C liposomes on the copper grids were observed using TEM.

The high stability and pH-sensitivity of the PC-C liposomes were directly observed by using TEM images. PC-C liposomes were collected after incubation at pH 7.4 and 5.0 for 24 h and prepared for TEM observation by negative staining using 1% phosphotungstic acid (PTA) solution. The TEM images are shown in Figure 4. The PC-C liposomes incubated at pH 7.4 for 24 h are depicted in Figure 4a; the structure of the PC-C liposomes could still be observed, which indicated that the PC-C liposomes possessed high stability. The PC-C liposomes incubated at pH 5.0 for 24 h are depicted in Figure 4b, and were found to be deformed and decomposed. The TEM results provided further evidence of the high stability and pH-sensitivity of the PC-C liposomes. To further clarify the mechanism of the pH sensitivity, the zeta potential was measured.

DPPC, PI, and all PC liposomal samples were incubated with pH 7.4 and pH 5.0 phosphate- buffered saline (PBS) at 37 °C for 6 and 24 h. Thereafter, the superficial zeta potentials were measured to study the pH copolymers and performance. The zeta potential results are presented in Table 2.

The charges of the DL and DCL liposomes were almost neutral charges at pH 7.4, with zeta potentials of −1.94 and −1.55 mV, respectively. The zeta potential of the PI liposomes was −27.73 ± 1.88 mV; the zeta potentials of the PC liposomes were all slightly higher than the PI liposomes at approximately −21 mV. The change in surface charge after the addition of crosslinkers was perhaps due to the variation in the nature of the solvent [33]. After incubation for 6 h at pH 7.4, the zeta potentials remained almost unchanged. The zeta potentials of the PC liposomes were higher than those of PI liposomes, because the crosslinkers in the PC liposomes were positively charged. At pH 5.0, DPPC liposomes did not show any significant change in zeta potentials, even after the addition of crosslinkers. This indicated that the crosslinkers were unable to attach to the surface of the DPPC liposomes because of the neutral charges on the DPPC liposomes. In contrast, the zeta potentials of the PI liposomes at pH 5.0 increased to −8.67 ± 0.36 mV after incubation for 6 h, which was evidence of the protonation of the carboxylic groups in the mPEG-*b*-P(MAAc)_40_-chol copolymers. At 6 h post-incubation, all PC liposomal zeta potentials were between −4 and −6 mV at pH 5.0, higher than those at pH 7.4, because of the pH-responsive protonation of the copolymer.

The measurement of zeta potential permitted the complete determination of the mechanism of pH sensitivity. The size changes in different pH conditions and the TEM images suggested that the PC-C liposome was the best option for further use as a crosslinked polymer–liposome. Henceforth, PC-C liposomes are presented as the crosslinking polymer–liposomes (PC liposomes), and their properties were investigated and discussed in the following sections.

### 2.4. Drug Leakage Tests

To investigate the feasibility of polymer–liposomes in cancer therapy, the anticancer drug doxorubicin (Dox) was loaded into the polymer–liposomes. Dox-loaded polymer-incorporated liposomes (Dox-loaded PI liposomes) and Dox-loaded crosslinking polymer–liposomes (Dox-loaded PC liposomes) were prepared by using remote loading methods, and studied for their stability and pH sensitivity through drug leakage and drug release experiments.

**Figure 5:**
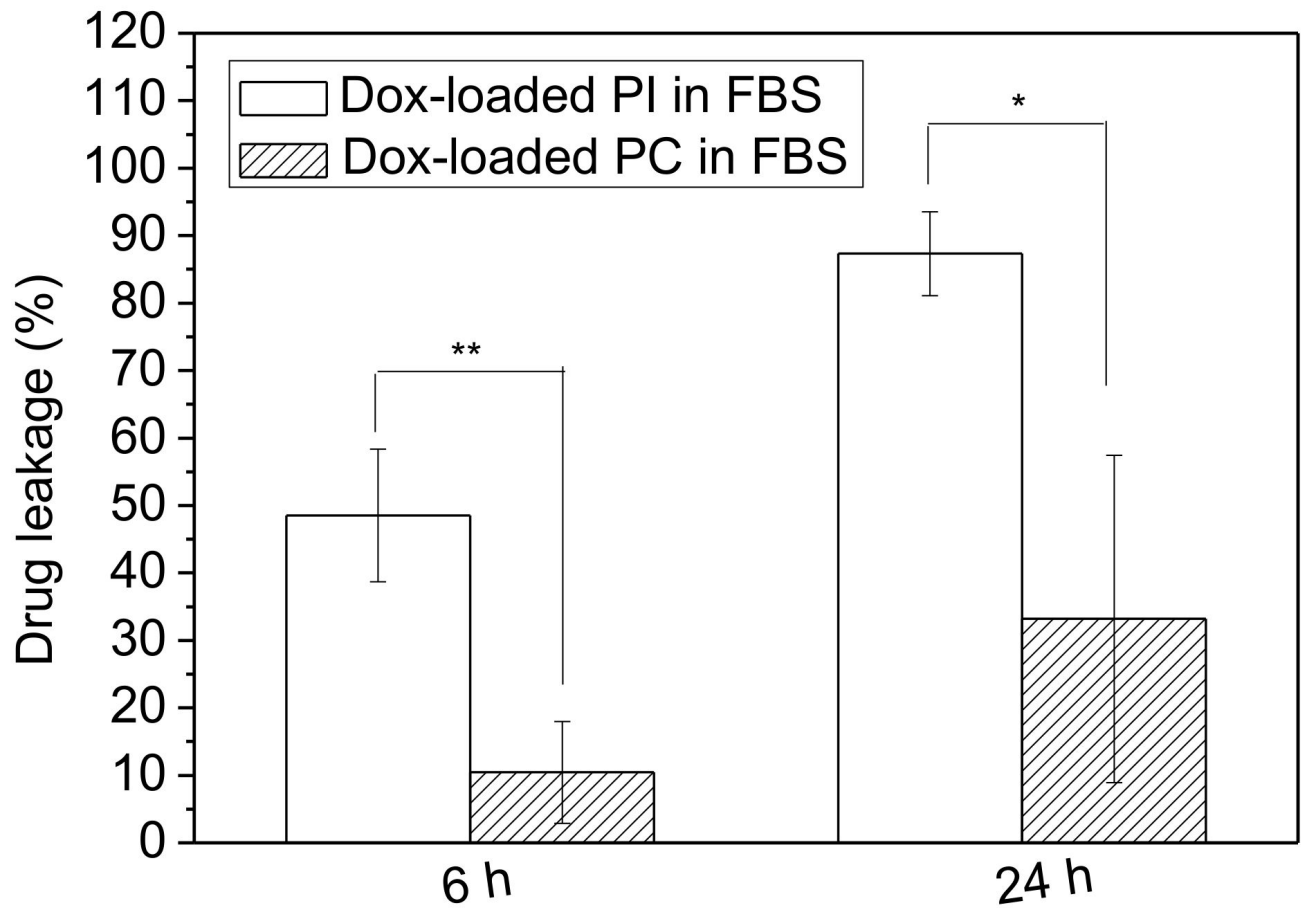
Drug leakage tests. Doxorubicin-loaded polymer-incorporated liposomes (Dox-loaded PI liposomes) and the doxorubicin-loaded crosslinking polymer–liposomes (Dox-loaded PC liposomes) were incubated in fetal bovine serum (FBS) at 37 °C, respectively. After 6 and 24 h, the released doxorubicin was detected by using UV–visible spectroscopy at 480 nm. The results were analyzed by using Student’s *t*-test in Microsoft Excel 2010. Significant differences are indicated with asterisks (* *p* < 0.05; ** *p* < 0.001).

To study the stability of the liposomes, Dox-loaded PI liposomes and PC liposomes were incubated with FBS to simulate the transportation process in blood circulation. The polymer–liposomes and FBS were thereafter placed in the dialysis bags and immersed in PBS at 37 °C and pH 7.4. At 6 and 24 h post-incubation, the released Dox was detected by using a UV–visible spectroscopy at 488 nm. The results are presented in Figure 5. After incubation for 6 h, 48% of the Dox was released from the PI liposomes; for the PC liposomes, only 10% of the Dox was released. After incubation for 24 h, 85% of the Dox was released from the PI liposomes; for the PC liposomes, only approximately 30% of the Dox was released. The leakage of Dox from PC liposomes is perhaps a result of dynamic disturbance within 6 h. The results clearly illustrated that the crosslinking networks enabled the stabilization of the polymer–liposomal structures, and therefore exhibited lower drug leakage.

### 2.5. Stability Tests of Dox-Loaded Polymer–Liposomes and Drug-Release Behaviors

**Figure 6:**
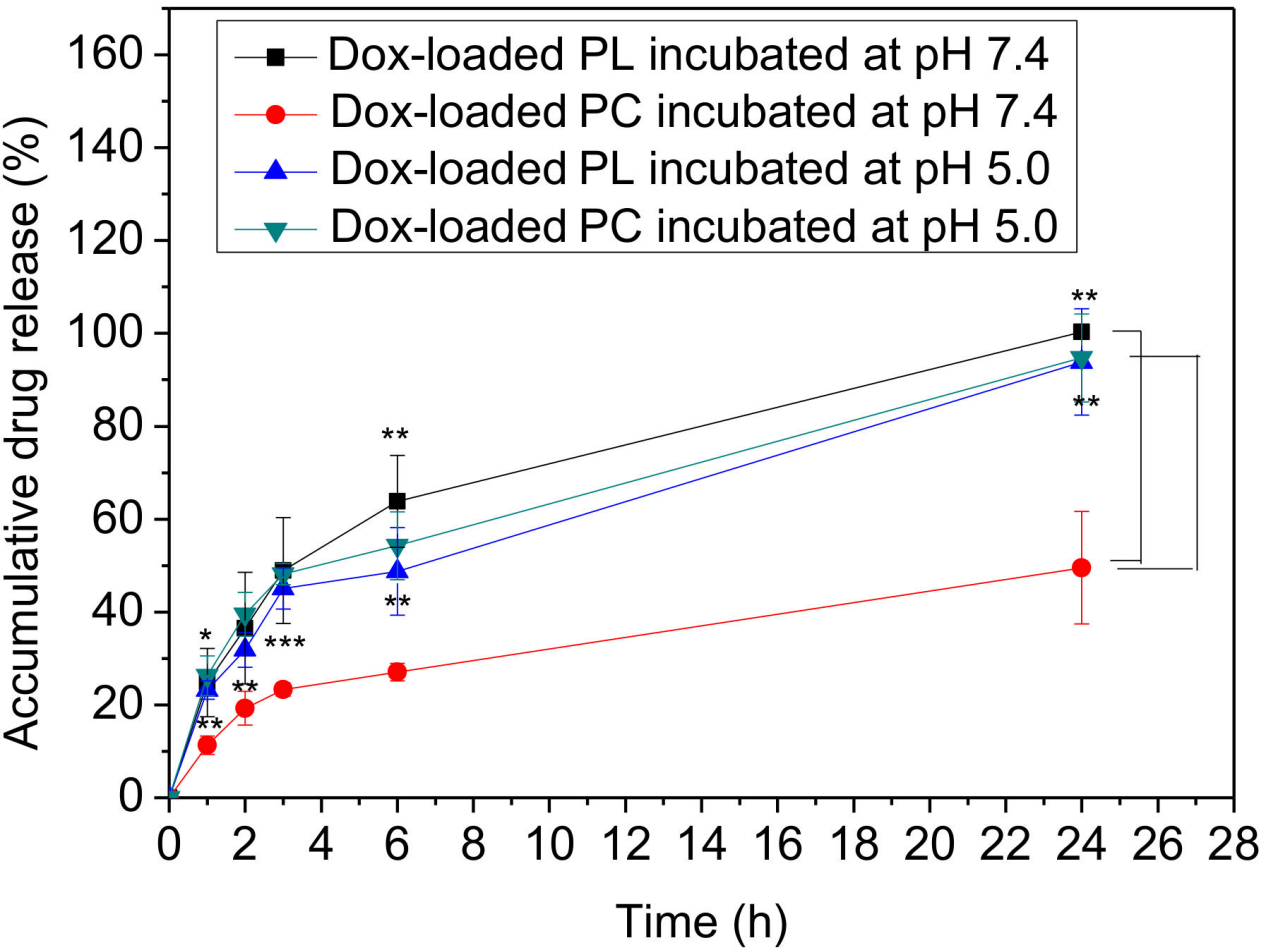
Drug releasing profiles. Anticancer doxorubicin loaded polymer–liposomes (Dox-loaded PL) and crosslinked polymer–liposomes (Dox-loaded PC) were incubated at pH 7.4 and pH 5.0. After incubation for 1, 2, 3, 6, and 24 h, the released doxorubicin was detected by using UV–visible spectroscopy at 480 nm. The data were compared by using Student’s *t*-tests in Microsoft Excel 2010. Significant differences are indicated with asterisks (* *p* < 0.05; ** *p* < 0.001; *** *p* < 0.0005).

To investigate the pH sensitivity of the PI and PC liposomes after loading of Dox; the Dox-loaded polymer–liposomes were placed into the dialysis bags and incubated at 37 °C in pH 7.4 and pH 5.0. After 6 and 24 h, free Dox was detected by using UV–visible spectroscopy at 480 nm. The results are shown in Figure 6.

As shown in Figure 6, Dox was released more slowly from Dox-loaded PC liposomes than from Dox-loaded PI liposomes at pH 7.4. After incubation for 24 h at 37 °C, the accumulated release rate of the PC liposomes was less than 50%, whereas PI liposomes released almost all the loaded Dox. The results corresponded to the particle size measurements and the findings of the drug leakage tests. The particle sizes of the PI liposomes increased with an increase in incubation time, whereas the particle sizes of the PC liposomes were unaltered. The increase in particle sizes revealed the instability of the liposomal structure; therefore, the encapsulated anticancer drug doxorubicin was released from the PI liposomes at pH 7.4. PC liposomes exhibited steady particle sizes at pH 7.4; therefore, they prevented the release of their payload.

The drug release results (Figure 6) further confirmed the pH-sensitivity of the polymer–liposomes. The PC liposomes released Dox more rapidly at pH 5.0 than at pH 7.4. After incubation for 1 h, only 10% of the encapsulated drug was released from the PC liposomes at pH 7.4, whereas more than 20% of the loaded Dox was released from the PC liposomes at pH 5.0. After 24 h, PC liposomes had released approximately 95% Dox in pH 5.0, whereas PC liposomes retained over 50% Dox at pH 7.4. However, it is difficult to assess the pH-responsive behavior of the PI liposomes, as their instability resulted in payload release at pH 7.4. The results indicated that crosslinkers have the ability to stabilize the polymer–liposomal structure and improve the stability of the polymer–liposomal system. The crosslinking reagents efficiently prevented drug leakage at pH 7.4, but preserved the pH-responsiveness of the Dox-loaded crosslinking polymer–liposomes.

In addition, to confirm the stability of the Dox-loaded polymer–liposomes, the particle sizes and PDI were monitored by using DLS; the results are shown in Figure S2a,b and displayed the particle sizes and PDI values of Dox-loaded PI liposomes and PC liposomes, respectively. The results indicated that the PI liposomes were larger than the PC liposome after doxorubicin loading. After incubation for 6 h, the size and PDI value of the Dox-loaded PI liposomes were increased two-fold. After 24 h, the Dox-loaded PI liposomes exhibited inhomogeneity in their size; however, the particle sizes of Dox-loaded PC liposomes were unchanged, although the PDI gradually increased over time. The results corresponded to the drug release profile in Figure 6. The Dox-loaded PI liposomes released 60% of their payload over 6 h at pH 7.4 and 37 °C, and almost all payload after 24 h owing to their instability. Dox-loaded PC liposomes maintained their particle size after incubation, which resulted in less drug release in equivalent conditions. The results further emphasized the role of the crosslinkers in polymer–liposomal stability.

### 2.6. Internalizations

After the successful transport and deposition of Dox-loaded PC liposomes in tumor lesions in the human body, the PC liposomes must still be internalized in cancer cells to achieve intracellular drug release. During endocytosis, endosomes and lysosomes form, and the environment in the endosomes and lysosomes gradually acidifies, reaching pH 5.0. The release rate of the encapsulated anticancer drug doxorubicin is an important requirement to achieve toxicity in cancer cells. The process of endocytosis and intracellular drug release was observed directly by using confocal laser scanning microscopy (CLSM).

To determine the localization of the Dox-loaded PC liposomes in the cells, hydrophobic fluorescent dye Cy 5.5 was first labeled onto the phospholipid bilayers. After the removal of excess fluorescent dye, human breast cancer cells (MDA-MB-231 cells) were treated with Cy 5.5-labeled Dox-loaded PC liposomes for 1 and 3 h. At 1 and 3 h post-incubation, the liposomes were removed, and the cells were washed three times with PBS. The cells were then stained with LysoTracker DND-Red to mark the endosomes and lysosomes; the nuclei were stained with 4′,6-diamidino-2-phenylindole (DAPI). The fluorescence of Cy 5.5, LysoTracker, doxorubicin, and DAPI are shown independently in grey, red, green, and blue.

**Figure 7:**
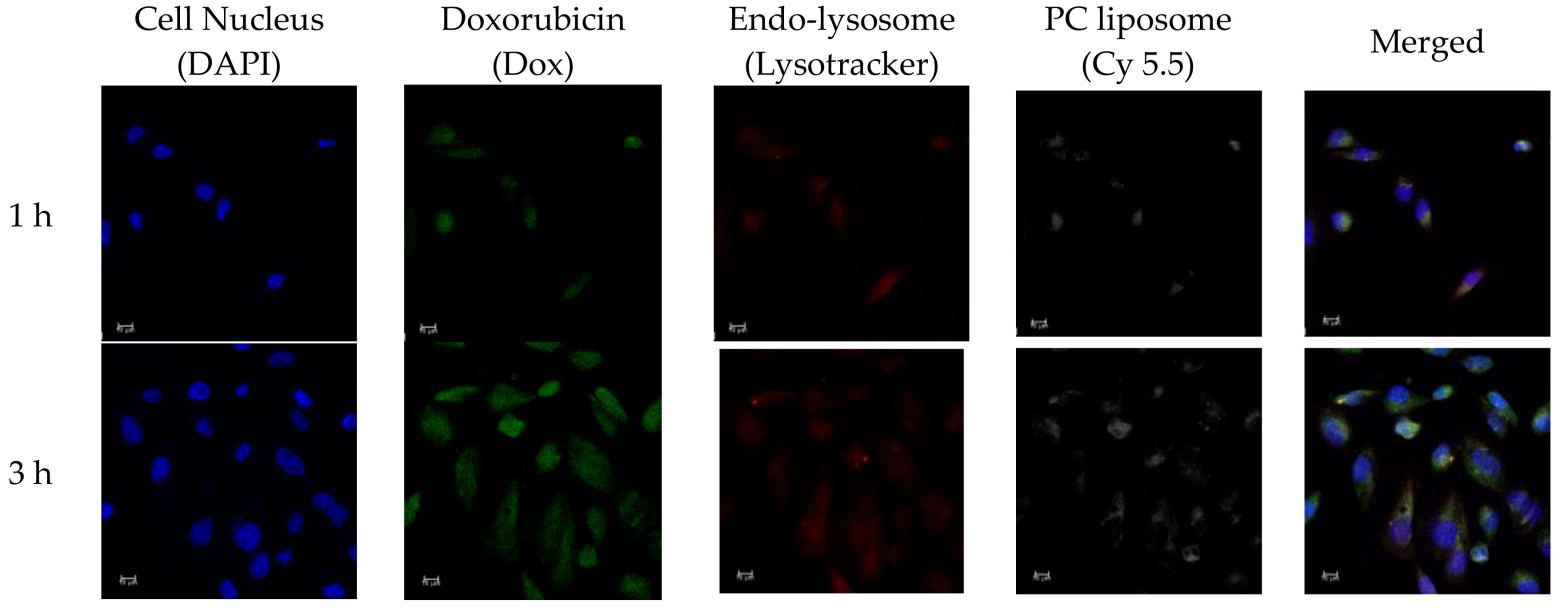
Internalization. Human breast cancer cells MDA-MB-231 were incubated with Cy 5.5-labeled Dox-loaded PC liposomes. After 1 and 3 h, PC-liposomes were removed, and the endolysosomes were stained by using LysoTracker Red DND-99. The cells were fixed in 4% paraformaldehyde solution and mounted by using DAPI-contained mounting medium. The fluorescence of the Cy 5.5-labelled Dox-loaded PC liposomes and MDA-MB-231 cells were observed by using confocal laser scanning microscopy (CLSM). The fluorescence of the Cy 5.5 and the anticancer drug Dox were excited, respectively, at 633 and 488 nm, and their fluorescences were observed at 650 and 520 nm, and shown in grey and green. The nucleus and endolysosomes of the MDA-MB-231 cells were marked and observed by using DAPI and 577/590 nm (excitation/emission) detecting conditions, respectively, shown in blue and red. The scale bar is 10 μm.

The CLSM images of the cells after incubation for 1 and 3 h are shown in Figure 7. These images demonstrated that the Cy 5.5-labeled Dox-loaded PC liposomes were facilitated into cancer cells by the incremental fluorescence of the PC liposomes. In addition, after incubation for 3 h, the Dox fluorescence increased and overlapped with the fluorescence of the lysosomes and PC liposomes, which demonstrated that doxorubicin was released from the PC liposomes within lysosomes and that intracellular drug delivery was achieved. The PC liposomes were endocytosed into cancer cells over time; moreover, they possessed pH-sensitivity that enabled the rapid release of their payloads in acidic pH conditions. The PC liposomes consistently accumulated in cancer cells and rapidly released doxorubicin, showing the ability to efficiently induce toxicity to cancer cells.

### 2.7. Cytotoxicity

**Figure 8:**
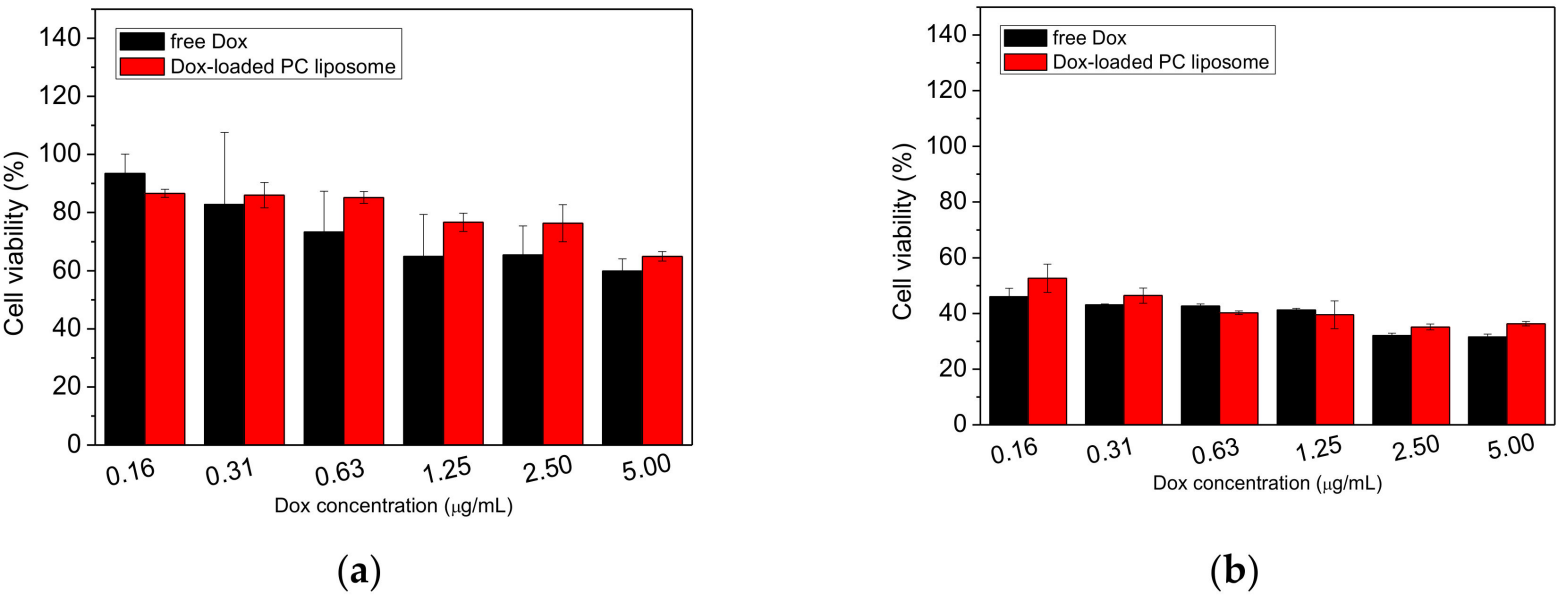
Cytotoxicity evaluation. The small molecule anticancer drug doxorubicin (Dox) and Dox-loaded PC liposomes were applied at the same Dox concentration to human breast cancer cells MDA-MB-231 cells and incubated at 37 °C in an atmosphere of 5% CO_2_ for 24 and 48 h. The excess free Dox and Dox-loaded PC liposomes were removed, and the cells were washed twice with PBS. The cell viability of the cells after 24 and 48 h incubation was determined using the MTT assay, respectively, and shown in (**a**,**b**).

As the PC liposomes were able to effect intracellular drug release, their cytotoxicity was also examined in this study by using an MTT assay. Dox-loaded PC liposomes and free doxorubicin were incubated with MDA-MB-231 cells at equivalent Dox concentrations. After 24 and 48 h, the cell viability of MDA-MB-231 cancer cells was measured by using the MTT assay; the results are shown in Figure 8a,b.

The viability of human breast cancer cells MDA-MB-231 after 24 h incubation with free doxorubicin and Dox-loaded PC liposomes is shown in Figure 8a. Both groups exhibited dose-dependent toxicity. The cell death increased with the applied Dox dose. After incubation for 24 h, the viability of the cells treated with 5 μg/mL of free Dox was 59%, whereas the viability of cells treated with 5 μg/mL of Dox-loaded PC liposomes was 64%. There was no significant difference between groups. The cytotoxicity of MDA-MB-231 cells after 48 h incubation is presented in Figure 8b. After incubation for 48 h, the half-maximal inhibitory concentration (IC_50_) for the treatment of free Dox to MDA-MB-231 cells was less than 0.16 μg/mL; the IC_50_ of the treatment of Dox-loaded PC liposomes was approximately 0.25 μg/mL. Although slightly lower IC_50_ was observed with free Dox, the different was not significant. The slightly lower IC_50_ of free Dox resulted the penetration of free Dox into the cancer cells via diffusion than endocytosis. Notably, the similar IC_50_ values indicated the rapid drug release properties of the Dox-loaded PC liposomes. The drug release profiles demonstrated the pH-sensitivity of the PC liposomes and CLSM images also showed that PC liposomes could be internalized into MDA-MB-231 cells and effect the intracellular release of doxorubicin. The cytotoxicity tests provide further evidence of the rapid drug release properties of PC liposomes.

## 3. Discussion

The stability of the pH-responsive polymer–liposomal system is a known drawback [29,30]. To achieve both high stability and pH-sensitivity, an electrostatically crosslinked polymer–liposomal system was developed. In this study, the pH-sensitive copolymer mPEG-*b*-P(MAAc)_40_-chol was synthesized and characterized, as shown in Figure 1. Poly(methacrylic acid) has been utilized in pH-sensitive nanoparticle drug delivery systems owing to its sensitivity at pH 5.0, which is close to the pH of the lysosomal environment [34,35]. The crosslinking reagent PEG-Lys2 was also synthesized and introduced into the polymer–liposomal system. The positively charged crosslinking reagents were able to interact with negatively charged copolymers; PEG-Lys2 formed an electrostatic network within the polymer–liposomes. Therefore, as shown in Table 2, the zeta potential of the polymer-incorporated liposomes was lower than that of the crosslinked polymer–liposomes, owing to the cationic crosslinking reagents. Lee et al. [31] previously reported polymer-caged liposomes, which were composed of covalent crosslinked bonds between pH-responsive copolymers. However, the polymer-caged liposomes exhibited slow drug release profiles. Less than 20% of the payloads were released within 24 h owing to the covalent bonds that irreversibly occupied the functional groups that were essential for pH sensitivity [31]. In this study, we utilized electrostatic interactions as the crosslinking modality, which were reversible, and did not affect pH sensitivity. While the cationic crosslinking reagents were considered toxic to cells at higher concentrations, the concentration of the crosslinking reagents was gradually increased until sufficient stability was achieved. The optimized results are shown in Figure 3a,b, and revealed that for a 16.85% molar ratio of crosslinking reagents, good stability of the polymer–liposomes was achieved.

The pH-sensitivity of the PC liposomes was also investigated. The particle sizes and distributions of the polymer-incorporated liposomes and the crosslinking polymer–liposomes at pH 5.0 are shown in Figure 3c,d. Apparent alterations of the particle sizes were observed. The pH sensitivity was attributable to the protonation of the carboxylic groups on mPEG-*b*-P(MAAc)_40_-chol copolymers, which was supported by the increased zeta potentials of the polymer–liposomes, as shown in Table 2. The results shown in Figure 3c also indicated that the PC liposomes exhibited obvious increments in particle sizes compared with the PI liposomes. The decomposition of the PC liposomes at pH 5.0 was also directly observed by using TEM (Figure 4). At pH 5.0, the crosslinking reagents remained positively charged, which would repel one another via electrostatic interaction and facilitate the decomposition of PC liposomes. The crosslinking reagents not only stabilized the polymer–liposomes, but may also enhance the pH sensitivity of the polymer–liposomes.

In addition, the stability and pH-sensitivity of the Dox-loaded PI and PC liposomes were measured, based on the drug leakage and release profiles (Figure 5 and Figure 6). Previous studies on poly(acrylic acid)-incorporated liposomes indicated that the instability of the polymer-incorporated liposomes was attributable to their anionic charges. In this study, the anionic copolymer mPEG-*b*-P(MAAc)_40_-chol also affected the stability of the Dox-loaded polymer-incorporated liposomes. The drug release profiles are shown in Figure 6; the Dox-loaded PI liposomes released 50% of their Dox payload within 6 h, which confirmed their instability and drug leakage under physiological conditions. The Dox-loaded PC liposomes were more stable than the Dox-loaded PI liposomes at pH 7.4. However, 30% of the Dox payload was released from the PC liposomes after 24 h incubation at 37 °C, because of the dynamic disturbances from the electrostatic interaction of the polymers. The PDI values of PC-C liposomes in Figure 3b confirmed the dynamic interference after incubation for 6 h at 37 °C. However, most of the leakage of Dox occurred within 6 h. In the presence of FBS, Dox-loaded PC liposomes still showed low drug leakage, which indicated that the crosslinking reagents assisted in the maintenance of the liposomal structures, as shown in Figure 5. Hioki et al. reported that 20% of Dox would be transduced from the commercial PEGylated liposomes in 6 h [36]. The electrostatic crosslinking polymer–liposomes leaked only approximately 10% of Dox in the presence of FBS within 6 h. In addition, the polymer-incorporated liposomes leaked more than 40% of the Dox within 6 h, which proved that the pH-responsive copolymers destabilized the liposomal structures. The results clearly demonstrated the high stability of the crosslinking polymer–liposomes, which helped to overcome the low stability problems of PEGylated liposomes and the polymer-incorporated liposomes in cancer therapy.

Based on the drug release profiles shown in Figure 6, the pH-sensitivity of the Dox-loaded crosslinking polymer–liposomes was retained, and resulted in rapid Dox release at pH 5.0, which was the simulated condition of the endolysosomes. Rapid drug release was observed in the endosomes or lysosomes within the human breast cancer cells MDA-MB-231 by using CLSM, as shown in Figure 7. In addition, the CLSM images in Figure 7 demonstrated that Dox-loaded PC-liposomes could be internalized into cancer cells after incubation for some time, despite the superficial negative charges of the crosslinking polymer–liposomes. In addition, Dox was released from the crosslinking polymer–liposomes dependent on the incubation time. Within 3 h of incubation, the fluorescence of Dox was observed in cancer cells, which indicated the release and distribution of Dox in the cells. The rapid drug release behavior led to rapid anticancer therapeutic effects. Herein, similar cytotoxic trends were noted for free Dox and Dox-loaded crosslinking polymer–liposomes, as shown in Figure 7. In our previous study, after co-culture with Dox-loaded polymer–liposomes in cancer cells for 48 h, the Dox-loaded polymer–liposomes exhibited a similar IC_50_ to free Dox. In this study, the cytotoxic effects appeared after incubation of MDA-MB-231 cells for 24 h with Dox-loaded crosslinking polymer–liposomes. The main cause may be the extra electrostatic repelling forces of the crosslinking reagents PEG-Lys2; therefore, faster drug release profiles were noted. The rapid intracellular drug release behaviors confirmed the anticancer efficiency.

The Dox-loaded crosslinking polymer–liposomes exhibited high stability and rapid intracellular drug release in simulated serum conditions, which represented an improvement in the two major challenges facing the use of polymer–liposomes. An excellent in vitro anticancer efficiency was also observed. The novel electrostatic crosslinking polymer–liposomes may be used in future anticancer therapies.

## 4. Materials and Methods

### 4.1. Materials

Methoxy poly(ethylene glycol) (mPEG) (*M*_W_ 5000), poly(ethylene glycol) (PEG) (*M*_W_ 400), dipalmitoylphosphatidylcholine (DPPC), cholesterol, 4,4′-azobis(4-cyanovaleric acid) (ABCPA), l-lysine (Lys), and phosphotungstic acid (PTA) were purchased from Sigma-Aldrich (St. Louis, MO, USA). Methacrylic acid (MAAc) and ammonium sulfate ((NH_4_)_2_SO_4_) were obtained from Acros Organics (Geel, Belgium). *N*-hydroxysuccinimide (NHS ester) was purchased from Alfa Aesar (Heysham, Lancashire, UK). Doxorubicin (Dox) was purchased from LC Laboratories (Woburn, MA, USA). Methanol and dichloromethane (DCM) were purchased from ECHO (Miaoli, Taiwan). The fluorescent dye cyanine 5.5-NHS ester (Cy5.5-NHS ester) was obtained from Lumiprobe (Hunt Valley, MD, USA). Fetal bovine serum (FBS) and LysoTracker Red DND-99 fluorescent dye were received from Thermo Fisher Scientific (Waltham, MA, USA). The PD-10 desalting column and Sephadex G 50 column were purchased from GE Healthcare Life Sciences (Little Chalfont, Buckinghamshire, UK).

### 4.2. Preparation and Characterization of pH-Responsive Polymer mPEG-b-P(MAAc)-chol

The macroinitiator mPEG2-ABCPA was prepared as previously reported [21]. Macroinitiator mPEG2-ABCPA (1 mmol) and the transferring reagent AET-HCl (1.2 mmol) were placed into a two-necked bottle and mixed with ethanol. To degas, inert gas (N_2_) was purged into the reacting system for 30 min, the monomer methacrylic acid (20 mmol) was then added under N_2_ into ethanol solution. The mixture was stirred and heated to 70 °C in an oil bath. After 24 h, the polymers were obtained after purification by ether precipitation. The polymers were thereafter dried at 60 °C in a vacuum oven. The dried polymers were weighed and dissolved into methanol with cholesterol-NHS ester (the molar ratio of polymer and cholesterol was 1:1.2), which was synthesized as per previously published protocols. After reaction for 24 h, the polymers were again purified by ether precipitation. The obtained polymers were dried in a vacuum oven at 60 °C and characterized using ^1^H NMR (Bruker NMR 500; DMSO-*d*_6_, Bruker, Rheinstetten, Germany) and FT-IR (Shimadzu FT-IR:IR Affinity-1; KBr, Shimadzu Corporation, Kyoto, Japan).

### 4.3. Preparation and Characterization of Crosslinker PEG-Lys2

PEG400 (*M*_W_ 400) was first modified with NHS-ester at the two end caps, as described by Wen et al. [37]. PEG-NHS ester2 (1 mmol) was weighed and dissolved in methanol with lysine (2 mmol). After reaction for 24 h with stirring at room temperature, PEG-Lys2 was purified by ether precipitation. The products were dried in a vacuum oven at 60 °C and characterized using ^1^H NMR (Bruker NMR 500; D_2_O) and FT-IR (Shimadzu FT-IR:IR Affinity-1; KBr).

### 4.4. Preparation and Characterization of Crosslinking Polymer–Liposomes

mPEG-*b*-P(MAAc)-chol and DPPC were weighed at various molar ratios, as summarized in Table 1. Polymers (5 mg) and DPPC lipids (10 mg) were independently dissolved in a mixture of DCM/methanol (1:1 *v*/*v*). The concentration of solute in DCM/methanol was approximately 5 mg/mL. The solvent was gradually removed by rotary evaporation and the polymer-incorporated lipid was formed. Subsequently, PBS at pH 7.4 (7.5 mL) was added to rehydrate the film. The concentration of solute in PBS was approximately 2 mg/mL. The polymer–liposomes were fabricated by sonication for 6 min and extrusion using a 0.22 μm PVDF filter and a 0.1 μm PVDF filter. To obtain crosslinked polymer–liposomes, the crosslinkers were added into the polymer–liposome solution and incubated for 2 h. The particle size and PDI of the polymer–liposomes were measured by using DLS (Malvern Zetasizer ZS90, Malvern Instruments, Worcestershire, UK) of a 0.1 mg/mL sample in PBS. The correlation function was analyzed by the CONTIN method (a constrained regularization method for inverting data).

### 4.5. Stability Tests and pH-Responsive Behaviors

The pH values of the polymer–liposome sample (including PI liposomes and all PC liposomes) in PBS were adjusted to 7.4 and 5.0 using 0.1 N HCl solution. The concentration of polymer–liposomes in PBS was approximately 0.1 mg/mL. The PI and PC liposomes were incubated at 37 °C with shaking (Taitec Bioshaker M-BR-022UP, Taitec, Tokyo, Japan). After 6 and 24 h, the particle sizes and distributions were measured by using DLS (Malvern Zetasizer ZS90). The morphologies of the polymer–liposomes were observed by using TEM (JEM-2000 EXII, Jeol, Tokyo, Japan). The polymer–liposome samples were dropped onto copper grids for 2 min after which the excess samples were removed. PTA (1% *w*/*v*) was dropped onto the copper grids for 1 min and the excess staining solution was removed. The TEM sample was dried and stored at room temperature. 

### 4.6. Zeta Potential Measurements

The PI and PC liposomes were mixed with PBS at a concentration of 0.1 mg/mL and the pH values were adjusted to 7.4 and 5.0 by using 0.1 N HCl solution, respectively. The concentration of polymer–liposomes in PBS was approximately 0.1 mg/mL. The PI and PC liposomes were incubated at pH 7.4 and 5.0 for 6 and 24 h. At the indicated time points, the PI liposome and PC liposomes (1 mL) were collected, transferred into a cuvette, and measured by using electrophoretic light scattering (ELS) (Malvern Zetasizer ZS 90).

### 4.7. Preparation of Dox-Loaded Crosslinking Polymer–Liposomes

Dox-HCl was encapsulated by using remote drug loading methods. mPEG-*b*-P(MAAc)-chol (5 mg) and DPPC (10 mg) were mixed in DCM/methanol solvents. The solvent was then evaporated and 250 mM (NH_4_)_2_SO_4_ (7.5 mL) was added to rehydrate the polymer–lipid film. The concentration of solute in (NH_4_)_2_SO_4_ solution was approximately 2 mg/mL. The polymer–lipid film was assembled into polymer-incorporated liposomes (PI liposomes) after sonication for 6 min and extrusion. The crosslinkers PEG-Lys2 were added into the PI liposomes to form the crosslinked polymer–liposomes (PC liposomes). The polymer–liposomes in (NH_4_)_2_SO_4_ were passed through Sephadex G 50 column using PBS as the mobile phase to produce loading gradients. Dox (3 mg), which was dissolved into PBS (0.3 mL) at 60 °C, was mixed with the PI and PC liposomes and the mixture was incubated at 60 °C for 2 h. Thereafter, the excess Dox was removed one further pass through the Sephadex G 50 column. The obtained Dox-loaded PI and PC liposomes were stored at 4 °C.

### 4.8. Drug Leakage in the Presence of Fetal Bovine Serum

Dox-loaded PI and PC liposomes in PBS (0.1 mg/mL) were mixed with an equal volume of 10% FBS in PBS. The mixture was placed into dialysis bags and was dialyzed in PBS at pH 7.4. The total volume of PBS was 5 mL. At specific time intervals (6 and 24 h post-incubation), the leakage of Dox was detected by using a UV–visible spectrum (PerkinElmer PDA UV/Vis Lambda 265, Perkin Elmer, Waltham, MA, USA) at 488 nm.

### 4.9. Stability Tests of Dox-Loaded Polymer–Liposomes and Drug Releasing Profiles

Dox-loaded PI and PC liposomes in PBS (50 μg/mL) were placed into dialysis bags (molecular weight cut-off 6–8 kDa). The dialysis bags were thereafter placed in PBS, whose pH values were adjusted to pH 7.4 and 5.0, and incubated at 37 °C. The total volume of PBS was 5 mL. At the indicated time points, PBS was collected and the released Dox concentration was determined by using a UV–visible spectrometer (PerkinElmer PDA UV/Vis Lambda 265) at the wavelength of 488 nm. In addition, the particle sizes of the Dox-loaded polymer–liposomes at pH 7.4 and 37 °C were also monitored by DLS to confirm the release behavior.

### 4.10. Internalization and Intracellular Drug Releasing Behaviors Observation

The fluorescent dye Cy 5.5-NHS ester was mixed with Dox-loaded PC liposomes in PBS at room temperature for 24 h to conjugate the amine groups to PEG-Lys2. The excess dyes were thereafter removed by using a PD-10 desalting column. The Cy 5.5-labeled Dox-loaded PC liposomes were obtained and stored at 4 °C. MDA-MB-231 human breast cancer cells were seeded onto 6-well plates at 3 × 10^5^ cells/mL. When the cells were attached to the 6-well plate, they were treated with the Cy5.5-labeled Dox-loaded PC liposomes. After 1 and 3 h, the Cy 5.5-labeled Dox-loaded PC liposomes were removed, and the cells were washed twice with PBS. The fluorescent dye lysotracker Red DND (1 μM) was co-cultured with the cancer cells for 2 h to stain the endosomes or lysosomes. Subsequently, excess fluorescence was eliminated, and the cells were washed twice with PBS. The cells were fixed with 4% paraformaldehyde solution for 20 min. After fixation, the cells were mounted with 4′,6-diamidino-2-phenylindole (DAPI)-containing mounting medium.

The intracellular drug release was observed by using a confocal laser scanning microscope (Leica SP8, Leica Microsystems, Wetzlar, Germany). The cells were stained with DAPI and observed under appropriate conditions for detection. To determine the localization of Cy5.5-labeled Dox-loaded PC liposomes, the fluorescence of Cy5.5 was observed at an excitation wavelength of 633 nm and an emission wavelength of 650 nm. The endosomes and lysosomes were localized by using LysoTracker Red DND and the fluorescence was detected by using 577 nm as the excitation wavelength and 590 nm as the emission wavelength. The fluorescence of Dox was observed at the excitation wavelength of 488 nm and at the emission wavelength of 520 nm. DAPI fluorescence was detected at an appropriate wavelength that was preset by the CLSM equipment [21,38].

### 4.11. Cell Cytotoxicity

Human breast cancer cells MDA-MB-231 were seeded onto a 96-well plate (7 × 10^3^ cells/mL). When the cells were attached, they were incubated with various concentrations of Dox HCl and Dox-loaded PC liposomes at 37 °C with 5% CO_2_ supply. After 24 and 48 h incubation, Dox and Dox-loaded PC liposomes were completely removed, and the cancer cells were washed twice with PBS. To the 96-well plate, 200 μL of MTT reagent (1 mg/mL) was added; after 2 h of treatment, the MTT reagent was removed by the addition of DMSO. The cell viability was determined by using an ELISA reader (Thermo Scientific Multiskan Go Microplate Spectrophotometer, Thermo Scientific, Vantaa, Finland) at the appropriate wavelength.

### 4.12. Statistical Analysis

All results represent the mean values and their standard deviations (mean ± SD). The comparisons were conducted with suitable references or control groups and analyzed by a two-tailed Student’s *t*-test (Excel, 2010, Microsoft, Washington, DC, USA). Differences were considered to be statistically significant for *p* values of less than 0.05.

## 5. Conclusions

pH-responsive polymer–liposomes have been considered a promising approach for the rapid release of payloads [15,23]. Roux et al. reported the difficulties in the formulation of a polymer–liposomal system with good stability as well as pH-responsive behavior [29]. In this study, highly stable and pH-sensitive crosslinking polymer–liposomes were designed and fabricated. A pH-sensitive copolymer PEG-*b*-P(MAAc)-chol and crosslinking reagents PEG-Lys2 were successfully synthesized and characterized for the first time. The pH-sensitive copolymers were introduced into DPPC lipid bilayers, forming polymer-incorporated liposomes, and the crosslinking reagents were also inserted into the polymers to form crosslinked polymer–liposomes. We optimized the degree of crosslinking based on the particle size measurement. The crosslinking polymer–liposomes were approximately 140 nm and the bilayer morphology was observed by using TEM. The crosslinking polymer–liposomes enabled not only the stabilization of the liposomal structure, but also were pH-sensitive. In the presence of serum proteins, the crosslinking polymer–liposomes exhibited high stability with no Dox leakage. In acidic conditions, they rapidly released their Dox payload. The rapid release behaviors also occurred in the intracellular environment. The CLSM images showed increased uptake of the crosslinking polymer–liposomes and Dox release over time. The crosslinked polymer–liposomes also exhibited cytotoxicity to the human breast cancer cell line MDA-MB-231. The crosslinked polymer–liposomes with electrostatic networks have overcome the stability problems associated with polymer–liposomes and retained the high pH sensitivity necessary for the rapid release of anticancer drugs within cancer cells. Therefore, they have potential clinical applications in the future. 

## Figures and Tables

**Table 1:** Components and particle sizes of the polymer-incorporated liposomes (PI liposomes) and the crosslinking polymer–liposomes (PC liposomes).

Code	Components (Molar %)			Particle Size (nm) *	PDI *
	Copolymer	Crosslinker	DPPC		
DL	0	0	100.00	144.27 ± 7.49	0.225 ± 0.029
DCL	0	15.6	84.35	151.10 ± 4.69	0.254 ± 0.022
PI	3.99	0	96.01	136.03 ± 2.71	0.200 ± 0.012
PC-A	3.80	4.82	91.38	139.57 ± 3.73	0.202 ± 0.007
PC-B	3.62	9.20	87.17	141.47 ± 1.91	0.199 ± 0.012
PC-C	3.32	16.85	79.83	141.10 ± 2.67	0.180 ± 0.001

* The particle sizes and particle dispersion index (PDI) were measured by using dynamic laser scattering (DLS).

**Table 2:** Zeta potentials of the DPPC and polymer–liposomes. The dipalmitoylphosphatidylcholine-liposomes (DPPC), polymer–liposomes (PI), and crosslinking polymer–liposomes (PC-A, PC-B, and PC-C) were independently incubated at pH 7.4 and pH 5.0 phosphate-buffered saline (PBS). After incubation for 6 h, the zeta potential was measured.

Code	ζ Potential at 0 h	ζ Potential at 6 h	
		pH 7.4	pH 5.0
DL	−1.94 ± 1.00	−4.25 ± 0.84	−1.96 ± 0.67
DCL	−1.55 ± 0.55	−7.07 ± 3.03	−2.33 ± 1.11
PI	−27.73 ± 1.88	−25.07 ± 1.96	−8.67 ± 0.36
PC-A	−21.77 ± 1.66	−22.00 ± 1.21	−6.20 ± 0.84
PC-B	−22.76 ± 0.67	−22.46 ± 1.31	−6.64 ± 0.48
PC-C	−22.70 ± 0.57	−21.03 ± 1.01	−4.78 ± 0.05

## Supplementary Materials

Supplementary materials can be found at http://www.mdpi.com/1422-0067/19/6/1615/s1.

## Abbreviations

PI liposomes	polymer-incorporated liposomes
PC liposomes	crosslinking polymer–liposomes
DPPC	dipalmitoylphosphatidylcholine
Dox	doxorubicin
