# Supplementary material for: Preparation and Characterization of Electrostatically Crosslinked Polymer–Liposomes in Anticancer Therapy
Source: Int J Mol Sci. 2018 May 30;19(6):1615. doi: 10.3390/ijms19061615 (PMC6032249; doi:10.3390/ijms19061615)
Supplement: Supplementary file 1 [file ijms-19-01615-s001.pdf]

Supplementary Materials: Preparation and Characterization of Electrostatically Crosslinked Polymer–Liposomes in Anticancer Therapy

Yi-Ting Chiang, Sih-Ying Lyu, Yu-Han Wen and Chun-Liang Lo

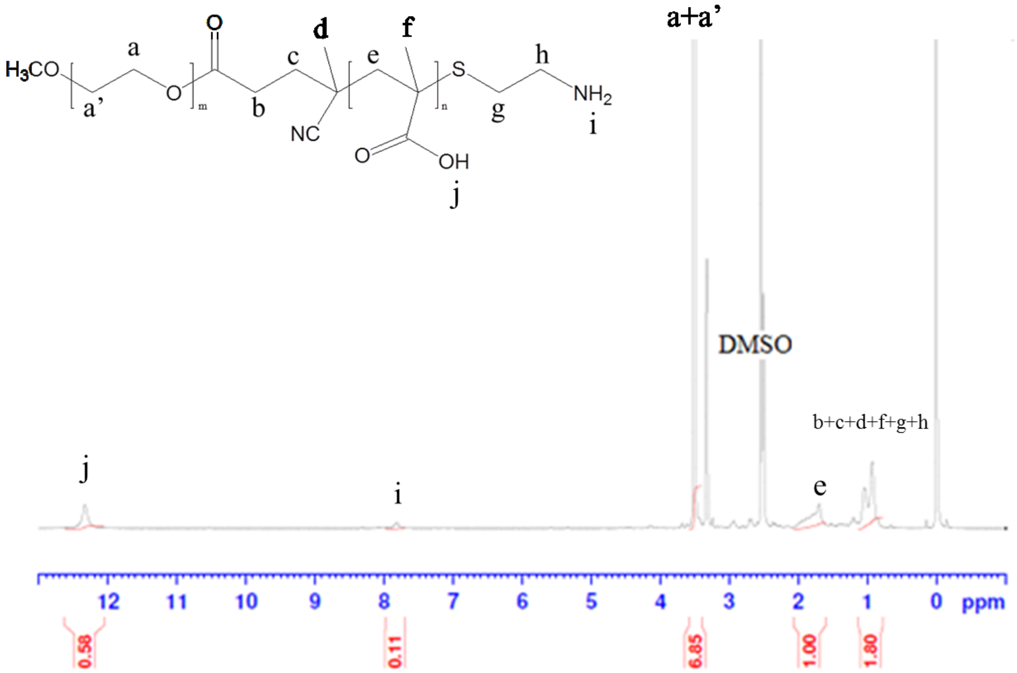

(**a**)

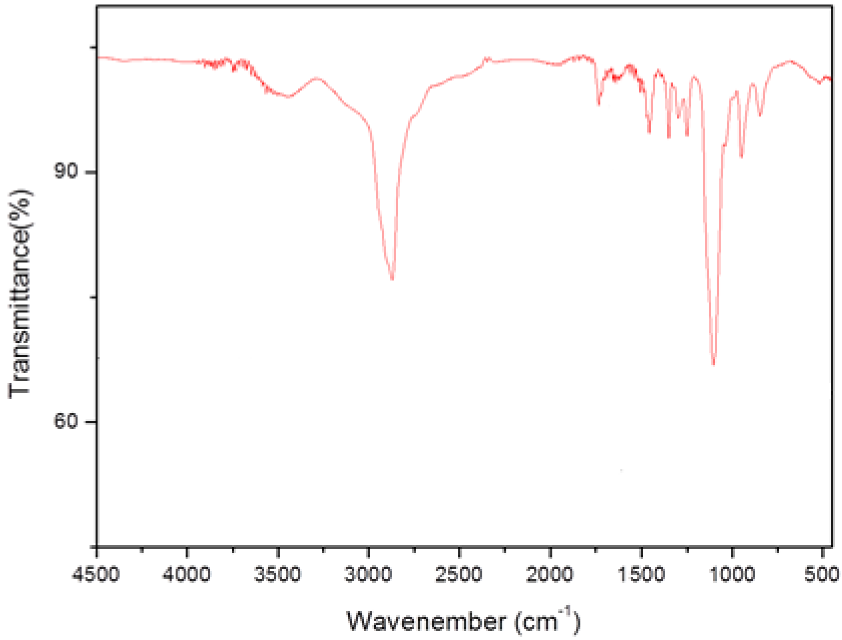

(**b**)

Figure S1. ^{1}H-NMR and FT-IR spectrum of the copolymer mPEG-*b*-P(MAAc)$_{40}$-NH$_2$. (**a**) The ^{1}H-NMR spectrum of the mPEG-*b*-P(MAAc)$_{40}$-NH$_2$; (**b**) The FT-IR spectrum of mPEG-*b*-P(MAAc)$_{40}$-NH$_2$.

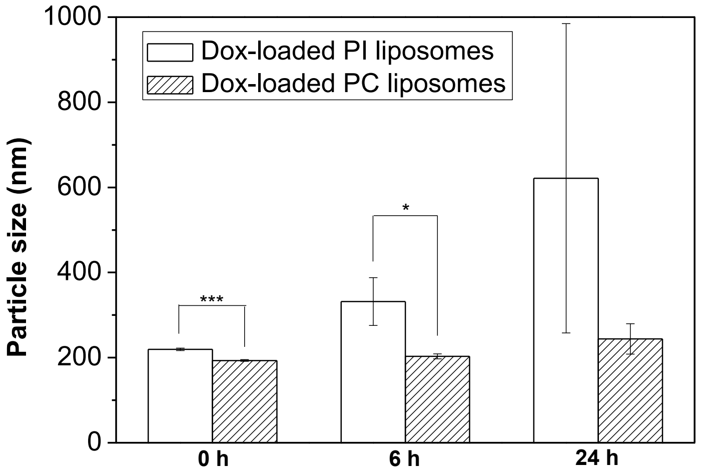

(**a**)

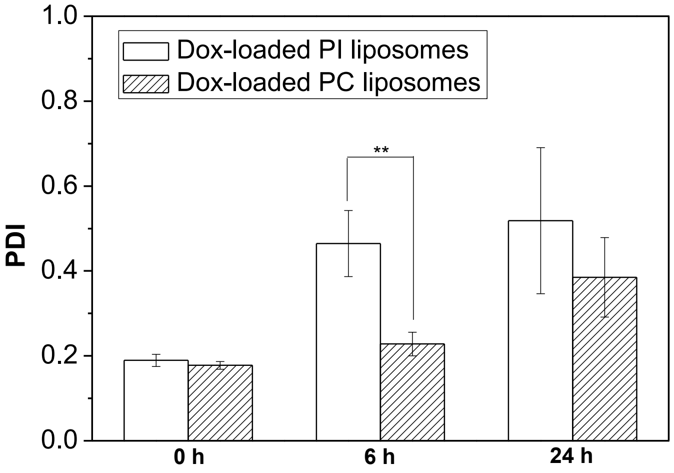

(**b**)

Figure S2. Stability of the Dox-loaded PI liposomes and PC liposomes. Liposomal samples were incubated at 37 °C pH 7.4 condition. After 6 and 24 h, the particle sizes and PDIs were measured using DLS. (**a**) The particle size of Dox-loaded polymer-liposomes; (**b**) The PDI of Dox-loaded polymer-liposomes. * $p < 0.05$; ** $p < 0.001$; *** $p < 0.0005$.
